# Composite risk stratification models optimise the value of imaging in prostate cancer staging

**DOI:** 10.1002/bco2.253

**Published:** 2023-05-21

**Authors:** Tom McAllister, Vincent Gnanapragasam, David Thurtle

**Affiliations:** ^1^ Clinical School University of Cambridge Cambridge UK; ^2^ Department of Surgery University of Cambridge Cambridge UK; ^3^ Cambridge University Hospitals NHS Foundation Trust Cambridge UK

**Keywords:** imaging, metastases, PCSM, prostate cancer, risk stratification, staging

The detection of distant metastases in prostate cancer (PCa) staging impacts upon prognosis and management options. CT scans and isotope bone scintigraphy (BS) remain the most widely used primary staging investigations, although PSMA PET is increasingly being used.^1^, ^2^ The UK National Institute for Health and Care Excellence (NICE) guideline update in 2021 recommended switching from a three‐tier system of risk stratification to the five‐tiered Cambridge Prognostic Groups (CPG).^3^ However, there is a lack of consensus across guidelines, on the optimal strategy for deciding which patients require, and who can reliably avoid, staging investigations. There is particular uncertainty in men with intermediate risk, or CPG3 disease—a question that NICE suggests requires further research.^4^ CT and BS are finite hospital resources, and attending for these can be a significant undertaking, may lengthen diagnostic pathways and increase anxiety. Increasingly, PSMA‐PET for primary staging may help replace these, but there is uncertainty about whether its use should be alongside conventional staging modalities.

In this study, we sought to compare different strategies to determine the most effective way to use primary staging investigations, considering both metastasis detection and the potential to safely omit imaging. Clinical records were retrieved for all patients presenting with apparent localised PCa, diagnosed by prostate biopsy in our tertiary centre between 2013 and 2020. Men presenting with metastases or PSA > 100 ng/mL were not included. The study was registered with Institutional Review Board approval (CUHNHSFT, UK [ID3458 PRN9458]).

The patient cohort was categorised using either individual attributes of (i) PSA at diagnosis (0 ≤ 10 ng/mL, 10 ≤ 20 ng/mL, 20 ≤ 30 ng/mL, 30 ≤ 50 ng/mL, 50+ ng/mL), (ii) MRI stage categorised into integer values (T1–T4), (iii) Grade Groups 1–5 or by composite prognostics/risk groups, (iv) Cambridge Prognostic Group system 1–5 and (v) the EAU three‐tier system based on D'Amico classification.^5^ These categories formed the basis for analysis of staging system performance.

Data from 783 men were available for analysis. Median age was 67 years (IQR 62–73) and PSA 7.5 ng/mL (IQR4.5–10.6). The cohort comprised 153 (19.5%) men with low‐risk, 329 (42.0%) with intermediate‐risk and 301 (38.4%) with EAU high risk disease. One hundred sixty‐five CT and 452 bone scans were performed across our cohort at diagnosis for primary staging. Within this cohort, 31 men (3.96%) had some form of detected metastasis: 13 had bone metastasis (1.66%), 12 had pelvic nodal disease (1.53%) and 6 had both (0.77%). Three hundred twenty‐two men (41.1%) had neither a staging CT nor BS, the majority of whom had early disease; 78.9% were CPG 1 or 2.

Staging thresholds of ≥CPG3 and ≥Intermediate risk would have ensured detection of all cases of metastases (NPV 100%). However, more scans could be avoided without missing any metastases, by using the CPG cut‐off. Indeed, avoiding CT or BS in any patient with CPG 1 or 2 disease would have avoided 104 unnecessary scans (16.9%) compared to 23 (3.73%) by avoiding scans in all EAU low‐risk cases. Specificity was therefore also superior using CPG3 at 18.3% compared to 4.1% for intermediate‐risk more broadly (Table 1). Specificity was increased further by using a threshold of ≥CPG4, which still detected all but one case of bone metastasis and all cases of nodal metastases in our cohort, correlating to an NPV of 99.1% and specificity of 40.1% overall. Using this threshold would have reduced the number of BS and CT performed by 188 (41.6%) and 42 (25.5%), respectively—a 37% reduction in the total number of scans. Of note, the CPG threshold for detecting nodal metastases could be higher than that for bone metastases in this cohort without missing any metastases. Using individual clinico‐pathological criteria such as Grade Group, MRI stage or PSA thresholds in isolation, however, provided inferior performance—with lower sensitivity and specificity and more unnecessary scans (Table 1).

**TABLE 1:** Summary statistics for each of the specified thresholds, looking at detection of metastasis by either computed tomography (CT) or bone scan (BS) staging.

	Imaging yields using individual risk attribute thresholds					Imaging yields using EAU risk group thresholds		Imaging yields using CPG group thresholds	
	MRI ≥ T2	MRI ≥ T3	Grade Group ≥ 2	PSA ≥ 10	PSA ≥ 20	≥Int risk	≥High risk	CPG ≥ 3	CPG ≥ 4
Total scans (CT + BS)	602	304	566	298	103	594	387	513	387
Sensitivity	100%	87.8%	95.9%	69.4%	44.9%	100%	95.9%	100%	95.9%
Specificity	2.6%	54.0%	8.6%	53.5%	85.7%	4.1%	40.1%	18.3%	40.1%
PPV	8.1%	14.1%	8.3%	11.4%	21.4%	8.2%	12.1%	9.6%	12.1%
NPV	100%	98.1%	96.1%	95.3%	94.7%	100%	99.1%	100%	99.1%
Scans avoided (%)	15 (2.4%)	313 (50.7%)	51 (8.3%)	319 (51.7%)	514 (83.3%)	23 (3.7%)	230 (37.3%)	104 (16.9%)	230 (37.3%)

*Note*: Separate details on individual scan yields by categories are given in the supporting information.

Abbreviations: CPG, 5‐tier Cambridge Prognostic Group classification; EAU, European Association of Urology 3‐tier risk classification; NPV, negative predictive value; PPV, positive predictive value.

We demonstrate that the CPG criteria, in a modern cohort assessed with upfront MRI, can better inform the value of staging investigations compared to either a three‐tiered system or using individual disease parameters. Our results validate the current NICE guidance to omit distant staging investigations completely in patients with CPG 1 and 2 disease. Furthermore, based on our results, confining CT and BS to CPG ≥ 4 would allow for significant reductions of 25.5% for CT usage, and 42% for BS usage compared to our current local practice, yet maintain very high negative predictive values in excess of 99%. These results mirror findings from a recent study from the National PCa registry of Sweden, which similarly found that the actual proportion of men with CPG4 or 5 disease with distant metastases remains low.^6^

The relatively small number of patients in our cohort who had metastases at staging (31 men, 3.96%) coupled with the fact that 461 patients (58.8%) had at least one staging investigation highlights the need to refine scan usage. Previous paradigms, based upon clinical T‐staging and results of non‐targeted biopsies, should be challenged in the modern era. This study goes further than much of the published literature, by assessing the utility of CT to identify soft tissue in addition to reviewing bone metastases presence. This work demonstrates the benefit of incorporating MRI‐staging information into risk categorisation to direct decision‐making, building upon similar previous work on bone scans alone.^7^

We recognise that PSMA PET‐CT is increasingly being used in the setting of staging new PCa patients and may represent a better gold‐standard for assessing metastases. However, the costs and logistic difficulties with this technology remain problematic, and the prudent husbanding of this resource will be important. The work outlined herewith would suggest the threshold for its use should again be based upon a combined risk model, and given the low likelihood of detecting metastases should perhaps be set at ≥CPG4—although this will need further validation work given that PSMA has been reported to have 27% greater accuracy over conventional imaging in primary staging.^8^

Limitations to our study include its retrospective observational nature, the small absolute numbers of men with metastases, and we recognise that use of staging investigations above certain thresholds was not universal, such that reported sensitivity and specificity rates should not be extrapolated to other cohorts. Future studies should look at validating our results in a prospective cohort, or in comparison with PSMA‐PET, or further‐investigating thresholds to guide when to use novel nuclear medicine imaging techniques.

Using a composite risk stratification system optimally informs decisions about when to avoid staging investigations in PCa. We conclude that doing staging for anything other than CPG4‐5 disease has extremely low yields and could be abandoned.

## CONFLICT OF INTEREST STATEMENT

The authors declare there are no conflicts of interest.

## Supporting information


**Appendix S1.** Supporting Information
