# Supplementary material for: Composite risk stratification models optimise the value of imaging in prostate cancer staging
Source: BJUI Compass. 2023 May 21;4(5):501–3. doi: 10.1002/bco2.253 (PMC10447204; doi:10.1002/bco2.253)
Supplement: Supplementary file 1 — Appendix S1. Supporting Information [file BCO2-4-501-s001.docx]

**Appendix**

| **CPG** | **Total patients** | **Had CT** | **Ct +ve for bone** | **CT +ve for node** | **Had BS** | **BS +ve** | **Bone only** | **Nodal disease only** | **Bone and node** |
| --- | --- | --- | --- | --- | --- | --- | --- | --- | --- |
| **1** | 153 | 6 | 0 | 0 | 17 | 0 | 0 | 0 | 0 |
| **2** | 193 | 13 | 0 | 0 | 68 | 0 | 0 | 0 | 0 |
| **3** | 136 | 23 | 1 (4.35%) | 0 | 103 | 1 (0.97%) | 1 | 0 | 0 |
| **4** | 163 | 49 | 2 (4.08%) | 3 (6.12%) | 131 | 3 (2.29%) | 3 | 3 | 0 |
| **5** | 138 | 74 | 11 (14.9%) | 13 (17.6%) | 133 | 15 (11.3%) | 9 | 9 | 6 |

| **MRI** | **Total patients** | **Had CT** | **Ct +ve for bone** | **CT +ve for node** | **Had BS** | **BS +ve** | **Bone mets** | **Nodal disease** | **Bone and node** |
| --- | --- | --- | --- | --- | --- | --- | --- | --- | --- |
| **T1** | 41 | 2 | 0 | 0 | 13 | 0 | 0 | 0 | 0 |
| **T2** | 505 | 63 | 2 (3.17%) | 1 (1.59%) | 235 | 3 (1.28%) | 3 | 1 | 0 |
| **T3** | 227 | 92 | 10 (10.9%) | 9 (9.78%) | 194 | 13 (6.70%) | 9 | 6 | 4 |
| **T4** | 10 | 8 | 2 (20%) | 6 (60%) | 10 | 3 (30%) | 1 | 5 | 2 |

| **Gleason** | **Total patients** | **Had CT** | **CT +ve for bone** | **CT +ve for node** | **Had BS** | **BS +ve** | **Bone mets** | **Nodal disease** | **Bone and node** |
| --- | --- | --- | --- | --- | --- | --- | --- | --- | --- |
| **3+3** | 198 | 13 | 1 (7.69%) | 0 | 38 | 1 (2.63%) | 1 | 0 | 0 |
| **3+4** | 301 | 39 | 2 (5.13%) | 1 (2.56%) | 153 | 2 (1.31%) | 2 | 1 | 0 |
| **3+5** | 15 | 4 | 0 | 0 | 14 | 1 (7.14%) | 1 | 0 | 0 |
| **4+3** | 132 | 35 | 0 | 2 (5.71%) | 117 | 1 (0.855%) | 1 | 2 | 0 |
| **4+4** | 30 | 11 | 0 | 0 | 27 | 0 | 0 | 0 | 0 |
| **4+5** | 73 | 41 | 5 (12.2%) | 8 (19.5%) | 71 | 8 (11.3%) | 4 | 6 | 4 |
| **5+3** | 2 | 1 | 0 | 0 | 2 | 0 | 0 | 0 | 0 |
| **5+4** | 26 | 17 | 4 (23.5%) | 4 (23.5%) | 24 | 4 (16.7%) | 2 | 2 | 2 |
| **5+5** | 6 | 4 | 2 (50%) | 1 (25%) | 6 | 2 (33.3%) | 2 | 1 | 0 |

| **PSA** | **Total patients** | **Had CT** | **CT+ve for bone** | **CT +ve for node** | **Had BS** | **BS +ve** | **Bone mets** | **Nodal disease** | **Bone and node** |
| --- | --- | --- | --- | --- | --- | --- | --- | --- | --- |
| **<10** | 526 | 71 | 5 (7.04%) | 5 (7.04%) | 248 | 5 (2.02%) | 3 | 3 | 2 |
| **10 - <20** | 186 | 56 | 2 (3.57%) | 5 (8.93%) | 139 | 5 (3.60%) | 4 | 5 | 1 |
| **20 - <30** | 44 | 17 | 2 (11.8%) | 0 | 40 | 3 (7.50%) | 2 | 0 | 1 |
| **30-<50** | 17 | 12 | 3 (25%) | 4 (33.3%) | 15 | 3 (20%) | 1 | 2 | 2 |
| **50+** | 10 | 9 | 2 (22.2%) | 2 (22.2%) | 10 | 3 (30%) | 3 | 2 | 0 |

| **EAU** | **Total patients** | **Had CT** | **CT +ve for bone** | **CT+ve for node** | **Had BS** | **BS +ve** | **Bone mets** | **Nodal disease** | **Bone and node** |
| --- | --- | --- | --- | --- | --- | --- | --- | --- | --- |
| **Low** | 153 | 6 | 0 | 0 | 17 | 0 | 0 | 0 | 0 |
| **Intermediate** | 329 | 36 | 1 (2.78%) | 0 | 171 | 1 (0.585%) | 1 | 0 | 0 |
| **High** | 301 | 123 | 13 (10.6%) | 16 (13.0%) | 264 | 18 (7.52%) | 12 | 12 | 6 |

Summary statistics for the cohort based upon CPG group, MRI stage, Gleason grade, PSA category and EAU risk group. Computed tomography (CT); Bone scan (BS); European Association of Urology (EAU) Cambridge Prognostic Group (CPG) classification.
